# Psychometric Properties of the Fear of Progression Questionnaire for Children in Two German Samples (Acute Treatment and Follow‐Up Care)

**DOI:** 10.1002/pon.70254

**Published:** 2025-08-13

**Authors:** Florian Schepper, Anja Santel, Jessy Herrmann, Kristina Herzog, Leonard Konstantin Kulisch, Jörn‐Sven Kühl, Julia Martini

**Affiliations:** ^1^ Department of Paediatric Oncology Haematology & Haemostaseology University of Leipzig Leipzig Germany; ^2^ Department of Paediatric Psychiatry, Psychotherapy, and Psychosomatics University of University Leipzig Germany; ^3^ Registered Association for Parents of Children with Cancer Leipzig Leipzig Germany; ^4^ Mental Health Research and Treatment Centre (FBZ) Faculty of Psychology Ruhr‐University Bochum and German Center for Mental Health (DZPG) Partner Site Bochum/Marburg Bochum Germany; ^5^ Department of Psychiatry and Psychotherapy Faculty of Medicine of the Technische Universität Dresden Dresden Germany

**Keywords:** cancer, children, factor analysis, fear of progression, oncology, psychometrics, quality of life, questionnaire, screening, validation

## Abstract

**Background:**

Fear of progression (FoP) is among the most prevalent psychosocial burdens in paediatric oncology. Empirical evidence suggests that FoP is particularly pronounced during acute treatment and often persists into follow‐up care. It is associated with heightened perception of physical symptoms, increased post‐traumatic stress symptoms, more negative illness perceptions, and reduced quality of life (QoL). Consequently, a comprehensive diagnostic instrument is essential to assess FoP in children with cancer.

**Objective:**

This study aimed to prospectively validate the German self‐report version of the Fear of Progression Questionnaire for Children (FoP‐Q‐SF/C) aged 7–18 years both during acute treatment and in follow‐up care.

**Methods:**

A total of 116 children participated in the study, including 39 undergoing acute treatment and 77 in follow‐up care. Participants completed the FoP‐Q‐SF/C. Factor analyses were conducted, and associations between FoP and QoL, illness perceptions, posttraumatic stress symptoms, time since diagnosis, cancer type and treatment modality were examined.

**Results:**

All items were applicable in children. The FoP‐Q‐SF/C demonstrated high internal consistency and good convergent, criterion and divergent validity. Consistent with previous research, factor analysis supported a one‐factor structure, although findings suggest potential for structural refinement.

**Conclusions:**

The FoP‐Q‐SF/C is a reliable and valid questionnaire for assessing FoP in children with cancer across different treatment phases. It serves as a valuable screening instrument to identify children at risk for elevated psychosocial distress and to guide targeted psychosocial interventions.

## 1 Background

Fear of progression (FoP) as well as the related construct fear of recurrence (FCR; [1]) are main psychosocial burdens in adult [2] and paediatric patients [3, 4] and relatives [5, 6, 7, 8]. These constructs describe the fear that a severe, potentially life‐threatening or incapacitating illness might recur or progress [1, 9]. FoP/FCR should be distinguished from psychiatric anxiety disorders, as they are based on the experience of a real threat [9]. Lower levels of FoP/FCR may not be dysfunctional, as they might help with adherence and compliance to medical procedures. Yet, elevated levels might negatively affect quality of life (QoL; [10]) or social functioning [2, 9].

Studies examining FoP/FCR in paediatric cancer patients used a wide range of different instruments: First studies applied an unvalidated single item to assess FCR [11]. Later, the Cancer Worry Scale was used to measure FCR in childhood cancer survivors [12]. Tutelman et al. [4] validated a child and a parent version of the Fear of Cancer Recurrence Inventory (FCRI). Luz et al. [3] adapted the German version of the FoP‐Q‐SF/PR for assessing FoP in parents [5] to measure FoP in children and Zhang et al. [13] adapted this instrument to study a Chinese sample of children with cancer.

In this article we focus on the construct of FoP and the FoP‐Q, which is a well‐known instrument to measure FoP in adult oncology [14]. This questionnaire consists of 5 dimensions to capture different aspects of FoP. Based on the FoP‐Q, a short‐form (FoP‐Q‐SF) was developed using items of four out of the five original dimensions [15] and was adapted for relatives [5, 8] and paediatric patients [3]. All versions of the FoP‐Q‐SF have been examined in terms of test theory and showed satisfying results regarding reliability and validity [3, 5, 8, 16]. All previous dimensionality analyses revealed two or more factors with eigenvalue > 1 but based on further analyses (e.g. visual testing via scree plot, parallel analyses) researchers opted for a one‐factor structure.

Levels of FoP have been shown to be associated with various medical factors: Previous research examined the relationship between FoP and time since diagnosis and found no significant results in paediatric patients [3, 17] and inconsistent results in their parents [5, 18, 19, 20]. Some evidence suggests that FoP may be more pronounced during the acute phase of treatment compared to follow‐up care [17, 18]. Concerning types of cancer and treatment, Zhang et al. [13] examined that FoP in children is predicted by having a reproductive system tumour and Peikert et al. [19] found higher levels of FoP in parents of children with a central nervous system tumour (CNS tumour) in comparison to leukaemia, possibly due to more frequent permanent restrictions caused by the treatment. However, in adult oncology no relation has been found between different types of cancer as well as treatment and the level of FCR [2].

Further research showed that the more physical symptoms parents perceive in their children with cancer, the higher is the parents' FoP [5]. There are similar findings for adult cancer survivors who had higher levels of FoP in relation to more perceived physical symptoms [21]. This shows that medical side effects of the treatment should be mitigated as much as possible and that psychosocial treatment is essential in supporting the patients and their relatives, preventing potential FoP later on. In addition, higher FoP in young adult cancer patients was associated with more negative illness perceptions [22], indicating that patients who perceive more severe consequences of their cancer and have more negative emotional representations show elevated levels of FoP. Herzog et al. [23] showed that the perception of more negative consequences is associated with lower QoL in children. Recent evidence suggests furthermore, that elevated levels of FoP in acute treatment and follow‐up care are associated with multiple psychosocial burdens like higher levels of posttraumatic stress [2, 3, 5, 20] as well as reduced QoL [3, 5, 19].

So far, for paediatric oncology no consensus has been reached what level of FoP should be considered as dysfunctional and requiring treatment. Clever et al. [5] suggested a cut‐off ≥ 34 on the FoP‐Q/SF to differentiate functional from dysfunctional FoP and studies adopting this show prevalences between 48% and 75.1% [19, 20]. For children, a distinction between low, intermediate and high FoP based on scale level has been proposed and elevated levels of FoP were found in approximately 20%–40% of children with cancer [3, 13, 17], highlighting the need for an appropriate screening instrument to identify these children and provide adequate treatment.

The objective of the present study was to further examine the adaptation of the FoP‐Q‐SF for children (age 7–18 years; FoP‐Q‐SF/C) and its psychometric properties in a larger sample. Building upon the study of Luz et al. [3], we address the following hypotheses.

1. The items of the FoP‐Q‐SF/C are applicable for children.
2. All items measure a common latent factor fear of progression.
3. *Convergent validity*: Children's FoP‐scores correlate positively with bodily symptoms (IPQ‐R; KINDL‐R), post‐traumatic stress symptoms (CATS) and perceptions of the illness (IPQ‐R).
4. *Criterion validity*: Children's FoP‐scores correlate negatively with QoL (KINDL‐R).
5. *Divergent validity*: Children's FoP‐scores show no correlation with time since diagnosis, type of cancer and type of treatment.

## 2 Methods

### 2.1 Design and Procedure

The present study was part of a larger research project that was conducted between 2019 and 2022 and consisted of 2 parts: Study 1 was a prospective‐longitudinal study with two data points (T1 and T2) 1 year apart in acute treatment, while study 2 was a cross‐sectional study with one data point (T1) in follow‐up care. The project has been pre‐registered at the German Clinical Trials Register (DRKS‐00022034) and at the Open Science Foundation (https://osf.io/fuahc).

Children in acute treatment (study 1) were recruited in the acute wards for paediatric oncology at the university hospitals in Dresden and Leipzig, Germany. Children in follow‐up care (study 2) were recruited in the parents' associations in Dresden and Leipzig (Sonnenstrahl e.V. Dresden, Elternhilfe e.V. Leipzig). Inclusion criteria were (1) oncology patients aged 4–18 years (for the subsample used for this analysis: 7–18 years) with any oncological diagnosis and one parent, (2) first diagnosis at least 1 month ago (study 1) or first diagnosis at least two years ago (study 2). Exclusion criteria were (1) inability to understand questionnaire/interview (e.g. due to language barriers or cognitive impairment; assessed by psychosocial staff), (2) palliative care. After obtaining written informed consent, the children completed different questionnaires. In addition, information on sociodemographic and medical characteristics of child and parent was obtained from the parent. All families who met the inclusion criteria were asked to participate.

Two previous studies have used the same data [10, 23]. For the present study data from study 1 baseline assessment (T1, children in acute treatment) and the cross‐sectional study 2 (children in follow‐up care) were used. Only children with FoP‐Q‐SF/C responses available were included in the present study. Further, questionnaires to assess post‐traumatic stress symptoms (CATS), QoL (KINDL‐R) and illness perceptions (IPQ‐R) as well as medical information were used in this study.

### 2.2 Sample

The sample consisted of *N* = 116 children with *n* = 39 children in acute treatment and *n* = 77 children in follow‐up care aged 7–18 years. Socio‐demographic and illness characteristics are summarised in Table 1.

**TABLE 1:** Sample characteristics.

	Acute treatment (*n* = 39)	Follow‐up care (*n* = 77)
Gender (*n*, %)		
male	23 (59.97)	48 (62.34)
female	16 (41.03)	29 (37.66)
Age (*M*, SD)	12.69 (2.79)	13.51 (2.63)
Diagnosis (*n*, %)		
Leukaemia	8 (20.51)	31 (40.26)
Lymphoma	11 (28.21)	9 (11.69)
Tumour of the central nervous system	6 (15.38)	18 (23.38)
Other solid tumour^a^	14 (35.90)	19 (24.68)
Time since diagnosis in months (M, SD)	8.62 (18.91)	83.85 (44.99)
Treatment type (*n*, % ‐ multiple responses possible)		
Chemotherapy	36 (92.31)	70 (90.91)
Radiotherapy	5 (12.82)	20 (25.97)
Surgical measures	18 (46.15)	29 (37.66)
Bone marrow/stem cell transplant	2 (5.13)	10 (12.99)
Other^b^	1 (2.56)	10 (12.99)

^a^ for example, kidney tumour, bone tumour.

^b^ for example, immune therapy, proton beam irradiation.

### 2.3 Measures

#### 2.3.1 Fear of Progression

Children from the age of 7 years completed the adaption of the FoP‐Q [14] for children (FoP‐Q‐SF/C [3]). The FoP‐Q‐SF/C measures FoP with 12 items on a five‐point Likert‐scale ranging from 1 (never) to 5 (very often). The FoP score is calculated by summing the items, with higher scores indicating greater FoP. Previously, the FoP‐Q‐SF/C showed good internal consistency, convergent and discriminant validity [3]. In the present study, internal consistency for the FoP‐Q‐SF/C was good (*α* = 0.87).

#### 2.3.2 Posttraumatic Stress Symptoms

The CATS (Child and Adolescent Trauma Screening) is a short instrument to screen patients' posttraumatic stress symptoms based on the DSM‐5 [24]. Children who experienced at least one traumatic experience rate their symptom burden using 20 items on a Likert‐scale ranging from 0 (never) to 3 (almost always). The global score is calculated by summing the scores. The CATS showed good reliability in a previous analysis [24]. Internal consistency in the present study was good (*α* = 0.80).

#### 2.3.3 Quality of Life

The KINDL‐R [25] was used to measure the child's QoL. The questionnaire consists of seven dimensions: (1) physical well‐being, (2) emotional well‐being, (3) self‐esteem, (4) family, (5) friends, (6) daily functioning (school or pre‐school/nursery school), and (7) illness. The dimensions are measured using 24 items on five‐point Likert‐scales ranging from 1 (never) to 5 (always). The scores of the dimensions are the means of the respective items after any reverse scoring. For the present study, all scores were transformed to values between 0 and 100 with higher scores indicating higher QoL. The German KINDL‐R has shown good internal consistency and high convergent and construct validity [25]. In the present study, internal consistency for the KINDL‐R version for 7–18‐year‐olds was good (*α* = 0.86).

#### 2.3.4 Illness Perception

Illness perceptions of children and parents were assessed using the short German Illness Perception Questionnaire (IPQ‐R; [26]) which consists of seven dimensions: (1) timeline‐acute/chronic (duration), (2) timeline‐cyclical (perceived trajectory as constant or cyclical), (3) consequences (impact of the illness on their life), (4) personal control (patient's influence over the illness), (5) treatment control (influence of the treatment on the outcome), (6) illness coherence (comprehension of the illness), and (7) emotional representations (emotional impact). The IPQ‐R assesses the symptoms present with 14 dichotomous items (yes/no) and the other dimensions with 18 items on a five‐point Likert‐scale ranging from 1 (strongly disagree) to 5 (strongly agree). The dimension scores are calculated by summing the items after reverse scoring. Higher scores indicate more associated symptoms, more negative perception of chronicity, cyclicity, and emotional representations, and more positive perception of personal control, treatment control and illness coherence. Previously, the German IPQ‐R showed sufficient internal consistency with alpha values between 0.70 and 0.87 (except treatment control; [27]). The subscales we used showed acceptable internal consistency (symptom attribution *α* = 0.69, consequences *α* = 0.71, emotional representation *α* = 0.74).

### 2.4 Statistical Analysis

Statistical analyses were performed using R Version 4.2.2 [28]. The significance level was set to *α* < 0.05 for all analyses.

Missing values were handled according to the respective test manuals. If no recommendations were provided, no missing values were allowed. For the FoP‐Q‐SF/C, item and scale analyses were calculated. Reliability was calculated using Cronbach's alpha (internal consistency). An exploratory factor analysis (EFA) with varimax rotation was performed to examine the factor structure. Model fit was checked with a confirmatory factor analysis (CFA). To test convergent, criterion, and divergent validity, correlations between variables were calculated with Spearman's correlation coefficient and Pearson's Chi‐Squared test. For convergent validity the IPQ‐R subscales symptom attribution, consequences and emotional representation, the global score of the CATS and the subscale physical well‐being of the KINDL‐R were used. The global score and the subscale psychological well‐being of the KINDL‐R were used for criterion validity. For divergent validity medical data about time since the diagnosis, type of cancer and type of treatment were used. Correlation coefficients *r* ≥ 0.10 were classified as low, *r* ≥ 0.30 as medium, and *r* ≥ 0.50 as large [29]. To estimate the impact of missing values, we conducted additional sensitivity analyses to check how many missing values are allowed in the FoP‐Q‐SF/C if missing values are replaced with the respective mean value of the remaining items.

## 3 Results

### 3.1 Descriptive Analysis of the FoP‐Q‐SF/C

Inter‐item correlations ranged from *r* = 0.16 (items 5 and 11) to *r* = 0.64 (items 2 and 9). The mean inter‐item‐correlation (IIC) was *r* = 0.37. The part‐whole corrected item‐total correlation (discriminatory power) was ≥ 0.50 for 11 items (except item 5) and ≥ 0.60 for items 6, 7, 8, 9 and 12. All items showed a non‐normal distribution with floor effect. The mean score of the FoP‐Q‐SF/C was 26.36 with a range from 12 to 58 not following a normal distribution (*p* = 0.005); the SD was 9.56. Highest scores were found for item 6 (family member will get the same illness) and item 11 (worry about family if something happens). Lowest scores were found for item 1 (anxious when thinking about progress) and item 7 (dependent on strangers' help). The range of the Likert scale was fully utilized by the sample. Further item characteristics are displayed in Table 2.

**TABLE 2:** Item characteristics of the FoP‐Q‐SF/C (*N* = 116).

	Item	*n*	*M*	SD [range]	*r*		Factor loadings	Factor loadings	
						*h* ^2^ 2 factors	1 factor	Factor 1	Factor 2
1.	I become anxious when I think that my disease may progress.	116	1.72	0.92 [1–5]	0.58	0.38	0.58	0.37	**0.46**
2.	I am nervous prior to doctors' appointments or periodic examinations.	116	2.37	1.24 [1–5]	0.50	0.51	0.54	0.14	**0.71**
3.	I am afraid that I may have pain.	116	2.28	1.23 [1–5]	0.50	0.38	0.54	0.22	**0.58**
4.	I have concerns about being less productive at school because of my disease.	116	2.37	1.41 [1–5]	0.51	0.34	0.56	**0.53**	0.23
5.	When I am anxious, I have physical symptoms (e.g., rapid heartbeat, stomach ache, nervousness).	116	2.14	1.22 [1–5]	0.37	0.15	0.41	0.28	**0.29**
6.	The possibility that my family can be affected by the same disease as me disturbs me.	116	2.68	1.36 [1–5]	0.61	0.44	0.63	**0.57**	0.30
7.	It disturbs me that I may have to rely on strangers for activities of daily living.	116	1.78	1.08 [1–5]	0.62	0.43	0.65	**0.52**	0.39
8.	I am worried that at some point in my life I will no longer to be able to pursue my hobbies because of my disease.	116	2.09	1.37 [1–5]	0.70	0.56	0.74	**0.56**	0.46
9.	I am afraid of severe medical treatments in the course of my disease.	116	2.14	1.19 [1–5]	0.62	0.70	0.68	0.25	**0.81**
10.	I worry that the medication could damage my body.	116	2.18	1.21 [1–5]	0.56	0.41	0.60	**0.56**	0.27
11.	I worry about what will become of my family if something should happen to me.	116	2.47	1.30 [1–5]	0.56	0.47	0.60	**0.68**	0.15
12.	The thought that I might be absent from school because of the disease disturbs me.	116	2.15	1.24 [1–5]	0.63	0.66	0.66	**0.79**	0.13

*Note:* Translation of the items from Luz et al. [3]. The numbers in bold show the factor on which the item loads the highest.

Abbreviations: Factor, Factor loadings of the principal component analysis; *h* ^2^, communality; *M*, mean; *n*, number of observations; *r*, statistical power; SD, standard deviation.

### 3.2 Factor Analysis

Kaiser‐Meyer‐Olkin Test (*KMO* = 0.83) and the Bartlett's test of sphericity (*p* < 0.001) indicated that the data was suitable for conducting a factor analysis. The EFA yielded one factor with Eigenvalue > 1 which explained 36.64% of the variance. Visual testing of the factor solution via scree plot supported this result. Contrary, a parallel analysis with simulated and resampled data showed a two factors model explaining 44.54% of the variance. Due to substantial cross‐loadings, a clear allocation of the items to two different factors is only possible to a limited extent.

The confirmatory factor analysis (CFA) to prove model fit showed mixed results: *SRMR* (0.080) supported the model while *χ* ^2^‐Test (*χ* ^2^ (54) = 148.570, *p* < 0.001), *RMSEA* (0.123), and *CFI* (0.813) showed an unsatisfactory model fit.

### 3.3 Construct Validity

The results on construct validity (descriptive statistics, internal consistency and correlation with the FoP‐Q‐SF/C global score) are summarized in Table 3.

**TABLE 3:** Results on construct validity (descriptive statistics, internal consistency and correlation with the FoP‐Q‐SF/C global score, *N* = 116).

Scale	*n*	*M*	SD [range]	Alpha	FoP‐Q‐SF/C	
					*r*	*p*
Convergent Validity						
Bodily symptoms						
KINDL‐R: Physical well‐being	115	3.70	0.90 [1–5]	0.82	−41	< 0.001***
IPQ: Current symptom attribution	53	2.26	2.38 [0–8]	0.74	0.40	0.003**
Post‐traumatic stress symptoms						
CATS: Global score	97	13.10	7.47 [0–45]	0.80	0.61	< 0.001***
Illness perception						
IPQ: Consequences	90	8.99	2.99 [3–14]	0.71	0.37	< 0.001***
IPQ: Emotional representation	91	5.73	2.21 [2–10]	0.69	0.46	< 0.001***
Criterion Validity						
Quality of life						
KINDL‐R: Global score	108	3.88	0.49 [2–5]	0.86	−0.35	< 0.001***
KINDL‐R: Psychological well‐being	115	4.00	0.64 [2–5]	0.66	−0.23	0.012*
Divergent Validity						
Medical information						
Time since diagnosis	112				−0.16	0.096
				df	*χ* ^2^	*p*
Type of cancer				224	225.47	0.460
Type of treatment						
		Chemotherapy Radio therapy Surgical measures Bone marrow		32	28.74	0.632
				32	33.79	0.381
				32	24.81	0.814
				32	40.63	0.141

Abbreviations: alpha, Cronbach's alpha; CATS, Child and Adolescent Trauma Screen; df, degrees of freedom; FoPQ‐SF/C, Fear of Progression Questionnaire child version; IPQ‐R, Illness‐Perception‐Questionnaire; KINDL‐R, Health‐Related Quality of Life; *M*, mean; *n*, number of observations; *p*, significance level; *r*, Spearman's correlation; SD, standard deviation; *χ* ^2^, Pearson's Chi‐Squared test.

#### 3.3.1 Convergent Validity

A moderate positive correlation between FoP and bodily symptoms measured with the scales physical well‐being (KINDL‐R) and current symptom attribution (IPQ‐R) was found (KINDL‐R: *r* = −0.41, *p* < 0.001; IPQ‐R; *r* = 0.40, *p* = 0.003). Similarly, FoP and posttraumatic stress symptoms (CATS) correlated highly positively (*r* = 0.61, *p* < 0.001).

Further, moderate positive correlations between FoP with the perception of consequences (IPQ‐R; *r* = 0.37, *p* < 0.001) and emotional representations (IPQ‐R; *r* = 0.46, *p* < 0.001) were found.

#### 3.3.2 Criterion Validity

QoL (KINDL‐R global score) showed a moderate negative correlation with children's FoP (*r* = −0.35, *p* < 0.001). Likewise, the subscale psychological well‐being correlated negatively with children's FoP (*r* = −0.23, *p* = 0.012).

#### 3.3.3 Divergent Validity

No correlation with the FoP score was found for time since diagnosis (*r* = −0.16, *p* = 0.096) nor type of cancer (*χ* ^2^_[224]_ = 225.47, *p* = 0.460). Different treatment types were not associated with level of FoP: chemotherapy (*χ* ^2^ [30] = 28.74, *p* = 0.632), radio therapy (*χ* ^2^ [30] = 33.79, *p* = 0.381), surgical measures (*χ* ^2^ [30] = 24.81, *p* = 0.814) and bone marrow transplant (*χ* ^2^ [30] = 40.63, *p* = 0.141) compared to the other treatment types, respectively.

#### 3.3.4 Sensitivity Analysis

For the sensitivity analysis, full case, imputation of one item and imputation of two items were compared. A descriptive comparison of the factor analysis showed no differences in RMSEA, SRMR and CFI. Similarly, there was no difference in the item characteristics and in the comparison of the validity analysis using the Fisher *z*‐test. A more detailed presentation of the sensitivity analysis can be found in Supporting Information S1: Appendix 1.

## 4 Discussion

FoP is a highly prevalent psychosocial burden in childhood cancer patients and survivors. To our knowledge, this is the first study with sufficient sample size to validate a self‐report instrument to measure FoP in children aged 7 years and older. We found high internal consistency as well as good convergent, criterion and divergent validity of the FoP‐Q‐SF/C. In line with previous research factor analysis revealed a one‐factor solution, even though there is potential for structural refinement.

The high internal consistency of the examined measure in this study is similar to internal consistencies in previous studies that measured FoP in children [3] and adults [14] with the FoP‐Q‐SF. Discriminatory power was good for all items except item 5 (having physical symptoms) with moderate discriminatory power [31] which is in line with previous results also showing good discriminatory power for all items except item 5 [3].

The fears with highest scores were about family members getting the same illness as well as worries about the family if something happens which underlines the importance of their families for children [32]. Psychosocial support in paediatric cancer care therefore is family‐oriented and takes into account the whole family system [30]. Our clinical experience shows that children may feel less burdened if they see that their parents cope reasonably well and are supported in their coping. On the other hand, lowest levels of fear were reported regarding thinking about the progress of the illness and being dependent on strangers' help. According to related research, children have different time frames than adults [33]. Over time, they develop the ability to plan future events and along with this ability the focus moves more to the future [34]. Therefore, children may think less about the future and a possible progress of their illness. In general, children may be more dependent on the help of others (e.g. parents) and have a lower perception of being restricted through the illness [3].

In line with previous research, this study suggest that one global factor of FoP might be the best solution to identify children with a high burden. Nevertheless, the mixed results of the parallel analysis and the CFA indicate, that the structure of the questionnaire may not be optimal. Previous studies also extracted one factor for children [3], parents [5] and adult patients [16] even though two or three factors had eigenvalues > 1. The presence of multiple factors becomes clear upon investigation of the long form of the FoP‐Q which originally comprised five different aspects of FoP [14]. If assuming a two‐factors structure, factor 1 seems to include items that ask about impacts on the social environment while factor 2 seems to contain items about medical treatment. These two aspects of FoP require different psychosocial treatments (e.g. family‐oriented care vs. pain management) and their possible distinction should be considered by clinicians and in further studies.

The convergent validity of the FoP‐Q‐SF/C was examined via correlations with bodily symptoms, post‐traumatic stress symptoms and more negative illness perceptions. All relations showed significant moderate to high positive correlations with FoP except physical well‐being (KINDL‐R) showing a significant moderate negative correlation, which is consistent with the hypothesis that higher levels of FoP are associated with more bodily symptoms and therefore, less physical wellbeing. These results are in line with previous studies of parents of children with cancer and adult patients showing a relationship between bodily symptoms and levels of FoP/FCR [2, 5, 21]. Previous studies also revealed correlations between levels of FoP/FCR and post‐traumatic stress symptoms in adult patients [2, 21] and parents of children with cancer [5, 7, 20]. Similarly, the relation between more negative illness perceptions and levels of FoP in parents of children with cancer [23] and adult patients [22] could be replicated for children in the current study.

Criterion validity of the FoP‐Q‐SF/C was examined via correlations with QoL. The significant moderate negative correlation with KINDL‐R global score and the low but significant negative correlation with KINDL‐R psychological well‐being supports criterion validity. These results are in line with previous studies for children [3], parents of children with cancer [7, 23] and adult patients [2, 21].

To test divergent validity of the FoP‐Q‐SF/C, several medical information were consulted. For time since diagnosis, type of cancer and type of treatment, correlations were not significant. The lack of correlation with time since diagnosis is consistent with previous studies in paediatric [3, 17] and adult cancer patients [2], although time alone might not be the decisive factor influencing the mitigation of FoP, but rather treatment status [17, 18]. Nonsignificant correlations were also found for type of cancer [9, 21] and type of treatment [2, 9] for adult patients, but not for parents of children with cancer [19]. Despite consistent results of the lack of association between levels of FoP and type of treatment, this relation should be examined in further studies. In the current sample, over 90% of all patients had chemotherapy, by far the most common treatment while other treatment types were less frequent (e.g., immune therapy). Studies of children with different types of cancer showed that some types of treatments can lead to higher mortality [35]. More far‐reaching consequences of the type of cancer treatment might also lead to an increased fear of these effects. A recurrence or progression of cancer would lead to a renewed treatment with the feared consequences and therefore higher levels of FoP. Further, divergent validity could be examined with other specific fears. FoP differs by definition from other fears because patients are confronted with real fears [9]. For other anxiety disorders, the main characteristic is that these fears are irrational [9]. Specific psychiatric fears like the fear of heights or the fear of specific animals should therefore show no correlation with FoP.

## 5 Limitations

This study presents several strengths like an adequate sample size and data from different treatment settings. Despite these positive characteristics, the study is not without limitations. Patients were only included in the second part of the study (follow‐up care) if they did not have a recurrence during the time of the study. This might limit the variance in levels of FoP and lead to lower levels in the sample. Further, the recruitment of the sample was realized by the psychosocial team of the participating hospitals and parents' associations which may affect representativity. Additionally, the distribution of illnesses and types of treatment does not reflect the distribution in the population which may bias the results.

Further studies should include longitudinal data to investigate the course of FoP. In addition, FoP should be studied in different types of treatment and in other age groups to explore differences according to disease, treatment and cognitive development of the child.

### 5.1 Clinical Implications

Given that FoP is related to a reduced quality of life, more posttraumatic stress and more negative illness perceptions, it should be assessed regularly in acute treatment and follow‐up care. The highest fear levels were related to family concerns, emphasizing the importance of family‐oriented psychosocial care.

## 6 Conclusion

This study provides evidence for the psychometric quality of the FoP‐Q‐SF/C as a self‐report instrument to assess FoP in children with cancer aged 7 years and older. The findings highlight the clinical relevance of FoP in paediatric oncology and its associations with psychosocial burdens (e.g. posttraumatic stress symptoms). Future research should investigate the questionnaire's factor structure, explore FoP across different treatment types and age groups, and examine its development over time.

## Author Contributions

Conception and design of the study: Julia Martini, Florian Schepper. Data collection: Kristina Herzog. Data analysis and interpretation: Anja Santel, Florian Schepper, Jessy Herrmann. Preparation of the manuscript: Florian Schepper. Critical review and approval of the final manuscript as submitted: Florian Schepper, Anja Santel, Jessy Herrmann, Kristina Herzog, Leonard Konstantin Kulisch, Jörn‐Sven Kühl, Julia Martini.

## Ethics Statement

This study was performed in line with the principles of the Declaration of Helsinki. It was approved by the ethics committee of the Technische Universität Dresden (EK 514112015) and the Universität Leipzig (366/14‐ff).

## Consent

Informed consent was obtained from all individual participants included in the study and from the parents of children who participated in the study.

## Conflicts of Interest

The authors declare no conflicts of interest.

## Supporting information


Supporting Information S1

## Data Availability

The data that support the findings of this study are available from the corresponding author upon reasonable request.
