# Supplementary material for: Psychometric Properties of the Fear of Progression Questionnaire for Children in Two German Samples (Acute Treatment and Follow‐Up Care)
Source: Psychooncology. 2025 Aug 13;34(8):e70254. doi: 10.1002/pon.70254 (PMC12350204; doi:10.1002/pon.70254)
Supplement: Supplementary file 1 — Supporting Information S1 [file PON-34-e70254-s001.docx]

**Appendix 1: Sensitivity Analysis**

The sensitivity analysis examines the influence of different methods for handling missing values on the results of the main analyses.

For this purpose, all children who answered the FoP-Q-SF/C (*n*=124) were consulted. Of these, *n*=5 children had one missing item in the questionnaire and *n*=3 children had two missing items in the questionnaire.

We therefore differentiated between three analyses, a full case analysis with no missing values (*n*=116), an analysis with the imputation of one missing item per person with the mean value of the remaining items (*n*=121) and an analysis with the imputation of up to two missing items per person with the mean value of the remaining items (*n*=124). No child had more than two missing values, so we could not further investigate this.

To compare the three analyses, all main analyses were carried out with the three datasets. An ANOVA compared the model fit of the CFA to compare the results of the factor analyses. Via F-Test we compared the mean values of the FoP-Q-SF/C and further looked at the respective characteristic values of the item analyses. For the comparison of the validity analyses we used Fisher-*z*-tests for all Spearman correlations. Because it is not possible to perform Fisher-*z*-tests on χ²-tests, we here compared the results by hand.

Results of all analyses are displayed in the tables below.

**Table 1.** Factor analyses

|  | Full case | max. 1 missing item | max. 2 missing items |
| --- | --- | --- | --- |
| *KMO* | .85 | .03 | .83 |
| Bartlett test | χ²=540.89, *df*=66, *p*<.001 | χ²=547.10, *df*=66, *p*<.001 | χ²=559.11, *df*=66, *p*<.001 |
| Variance of factor | .37 | .37 | .36 |
| χ²-test | χ²=148.37, *df*=54, *p*<.001 | χ²=152.34, *df*=54, *p*<.001 | χ²=162.35, *df*=54, *p*<.001 |
| *SRMR* | .080 | .078 | .081 |
| *RMSEA* | .123 | .123 | .127 |
| *CFI* | .813 | .807 | .792 |

*KMO*: Kaiser-Meyer-Olkin, *SRMR*: Standardized Root Mean Square Residual, *RMSEA*: Root Mean Squared Error of Approximation, *CFI*: Comparative Fit Index

The ANOVA comparing the three models showed a difference in χ² between the full case model and the model with one item missing of χ²diff=-10.007 (*df*=0, *p*<.001) and between the full case model and the model with up to two items missing of χ²diff=13.979 (*df*=0, *p*<.001).

**Table 2.** Item analyses

|  | Full case | max. 1 missing item | max. 2 missing items |
| --- | --- | --- | --- |
| Mean inter-item correlation | .37 | .36 | .36 |
| Range inter-item correlation | .16 - .64 | .17 - .64 | .16-.66 |
| *M (SD)* | 26.36 (9.56) | 26.36 (9.52) | 26.40 (9.47) |
| Cronbach’s α | .87 | .87 | .87 |
| *F*-Test | *F*=0.001, *p*=.999 | | |

M: mean, SD: standard deviation

**Table 3.** Validation

| Scale | Full case | | | | max. 1 missing item | | | | max. 2 missing items | | | |
| --- | --- | --- | --- | --- | --- | --- | --- | --- | --- | --- | --- | --- |
|  | *r* | | *p* | | *r* | | *p* | | *r* | | *p* | |
| *Convergent Validity* | | |  | |  | |  | |  | |  | |
| **Bodily symptoms** | | |  | |  | |  | |  | |  | |
| KINDL-R: Physical well-being | -.41 | | <.001 | | -.42 | | <.001 | | -.43 | | <.001 | |
| IPQ: Current symptom attribution | .40 | | .003 | | .42 | | .001 | | .42 | | .001 | |
| **Post-traumatic stress symptoms** | | | | | | |  | |  | |  | |
| CATS: Global score | .61 | | <.001 | | .62 | | <.001 | | .60 | | <.001 | |
| **Illness perception** | | | | |  | |  | |  | |  | |
| IPQ: Consequences | .37 | | <.001 | | .37 | | <.001 | | .37 | | <.001 | |
| IPQ: Emotional representation | .46 | | <.001 | | .45 | | <.001 | | .45 | | <.001 | |
| *Criterion Validity* |  | |  | |  | |  | |  | |  | |
| **Quality of Life** |  | |  | |  | |  | |  | |  | |
| KINDL-R: Global score | -.35 | | <.001 | | -.37 | | <.001 | | -.37 | | <.001 | |
| KINDL-R: Psychological well-being | -.23 | | .012 | | -.26 | | .004 | | -.23 | | .009 | |
| *Divergent Validity* |  | |  | |  | |  | |  | |  | |
| **Medical Information** | | | | |  | |  | |  | |  | |
| Time since diagnosis | -.16 | | .096 | | -.16 | | .095 | | -.15 | | .103 | |
|  | Χ² | *df* | | *p* | Χ² | *df* | | *p* | Χ² | *df* | | *p* |
| Type of cancer | 225.47 | 224 | | .460 | 267.75 | 252 | | .237 | 260.75 | 252 | | .339 |
| Type of treatment |  |  | |  |  |  | |  |  |  | |  |
| Chemotherapy | 28.74 | 32 | | .632 | 37.54 | 36 | | .399 | 38.78 | 36 | | .345 |
| Radio therapy | 33.79 | 32 | | .381 | 38.05 | 36 | | .376 | 36.10 | 36 | | .464 |
| Surgical measures | 24.81 | 32 | | .814 | 29.40 | 36 | | .744 | 28.65 | 36 | | .803 |
| Bone marrow | 40.63 | 32 | | .141 | 47.11 | 36 | | .102 | 42.41 | 36 | | .214 |

*r*: Spearman’s correlation; *p*: significance level; FoPQ-SF/C: Fear of Progression Questionnaire child version; KINDL-R: Health-Related Quality of Life; IPQ-R: Illness-Perception-Questionnaire, CATS: Child and Adolescent Trauma Screen; *df*: degrees of freedom; *χ*²: Pearson’s Chi-Squared test

**Table 4.** Validation, comparison via Fisher-*z*-test

| Scale | Full case – max. 1 missing item | | Full case – max. 2 missing items | | max. 1 missing item – max. 2 missing items | |
| --- | --- | --- | --- | --- | --- | --- |
|  | *z* | *p* | *z* | *p* | *z* | *p* |
| *Convergent Validity* |  | |  | |  | |
| **Bodily symptoms** |  | |  | |  | |
| KINDL-R: Physical well-being | 0.172 | .864 | -0.266 | .790 | -0.09 | .925 |
| IPQ: Current symptom attribution | -0.133 | .894 | 0.133 | .894 | 0.00 | >.999 |
| **Post-traumatic stress symptoms** | | |  | |  | |
| CATS: Global score | -0.112 | .911 | -0.111 | .911 | -0.224 | .823 |
| **Illness perception** |  | |  | |  | |
| IPQ: Consequences | 0.019 | .985 | -0.030 | .976 | -0.011 | .991 |
| IPQ: Emotional representation | -0.049 | .961 | -0.101 | .919 | -0.151 | .880 |
| *Criterion Validity* |  | |  | |  | |
| **Quality of Life** |  | |  | |  | |
| KINDL-R: Global score | 0.142 | .887 | -0.144 | .886 | -0.001 | .999 |
| KINDL-R: Psychological well-being | 0.221 | .825 | -0.020 | .984 | 0.205 | .838 |
| *Divergent Validity* |  | |  | |  | |
| **Medical Information** | | |  | |  | |
| Time since diagnosis | -0.021 | .984 | 0.060 | .953 | 0.040 | .969 |

*z*: Fisher-*z*-test; *p*: significance level; FoPQ-SF/C: Fear of Progression Questionnaire child version; KINDL-R: Health-Related Quality of Life; IPQ-R: Illness-Perception-Questionnaire, CATS: Child and Adolescent Trauma Screen

The sensitivity analysis shows that the results are robust to different methods for handling missing values. Still, we decided to use only full cases for the main analyses to avoid potential biases if missing values are not completely at random and because this is the most transparent method for future users of the FoP-Q-SF/C.
